# A New Era of Therapeutic Strategies for Chronic Thromboembolic Pulmonary Hypertension by Two Different Interventional Therapies; Pulmonary Endarterectomy and Percutaneous Transluminal Pulmonary Angioplasty

**DOI:** 10.1371/journal.pone.0094587

**Published:** 2014-04-11

**Authors:** Takumi Inami, Masaharu Kataoka, Motomi Ando, Keiichi Fukuda, Hideaki Yoshino, Toru Satoh

**Affiliations:** 1 Division of Cardiology, Second Department of Internal Medicine, Kyorin University School of Medicine, Tokyo, Japan; 2 Department of Cardiology, Keio University School of Medicine, Tokyo, Japan; 3 Department of Cardiovascular Surgery, Fujita Health University, Aichi, Japan; VU University Medical Center, Netherlands

## Abstract

**Background:**

Pulmonary endarterectomy (PEA) is established for the treatment of chronic thromboembolic pulmonary hypertension (CTEPH). Recently, percutaneous transluminal pulmonary angioplasty (PTPA) has been added for peripheral-type CTEPH, whose lesions exist in segmental, subsegmental, and more distal pulmonary arteries. A shift in clinical practice of interventional therapies occurred in 2009 (first mainly PEA, later PTPA). We examined the latest clinical outcomes of patients with CTEPH.

**Methods and Results:**

This study retrospectively included 136 patients with CTEPH. Twenty-nine were treated only with drug (Drug-group), and the other 107 underwent interventional therapies (Interventions-group) (39 underwent PEA [PEA-group] and 68 underwent PTPA [PTPA-group]). Total 213 PTPA sessions (failures, 0%; mortality rate, 1.47%) was performed in the PTPA-group (complications: reperfusion pulmonary edema, 7.0%; hemosputum or hemoptysis, 5.6%; vessel dissection, 2.3%; wiring perforation, 0.9%). Although baseline hemodynamic parameters were significantly more severe in the Interventions-group, the outcome after the diagnosis was much better in the Interventions-group than in the Drug-group (98% vs. 64% 5-year survival, p<0.0001). Hemodynamic improvement in the PEA-group was a 46% decrease in mean pulmonary arterial pressure (PAP) and a 49% decrease in total pulmonary resistance (TPR) (follow-up period; 74.7±32.3 months), while those in the PTPA-group were a 40% decrease in mean PAP and a 49% decrease in TPR (follow-up period; 17.4±9.3 months). The 2-year survival rate in the Drug-group was 82.0%, and the 2-year survival rate, occurrence of right heart failure, and re-vascularization rate in the PEA-group were 97.4%, 2.6%, and 2.8%, and those in the PTPA-group were 98.5%, 2.9%, and 2.9%, respectively.

**Conclusion:**

The patients who underwent interventional therapies had better results than those treated only with drugs. The availability of both of these operative and catheter-based interventional therapies leads us to expect the dawn of a new era of therapeutic strategies for CTEPH.

## Introduction

Chronic thromboembolic pulmonary hypertension (CTEPH) is a progressive and life-threatening disease in which chronic thromboembolism in the pulmonary arteries leads to pulmonary hypertension and right heart failure [1]–[9]. Medical therapies using anticoagulation and therapies targeted to pulmonary arterial hypertension are mildly effective for the treatment of CTEPH [1], [10], [11].

Conventionally, surgical pulmonary endarterectomy (PEA) has been considered the first choice of treatment for CTEPH [12]–[16]. Feinstein et al suggested that balloon pulmonary angioplasty is feasible for the treatment of CTEPH [17], and in recent several years, our group and others have reported that repeated balloon pulmonary angioplasty, so-called percutaneous transluminal pulmonary angioplasty (PTPA), improved subjective symptoms and hemodynamic parameters in patients with peripheral-type CTEPH, whose thromboembolic lesions exist in segmental, subsegmental, and more distal pulmonary arteries. Moreover, PEA is rather invasive and has the issue of residual or recurrent pulmonary hypertension [18]. Residual pulmonary hypertension contributes to poor life expectancy in patients with CTEPH [18], [19]. Repeated PEA is not easy to perform in patients with residual pulmonary hypertension because of high perioperative risk. PTPA, which is safe and less invasive, may complement these drawbacks of PEA [20]–[26].

Therefore, the objective of this study was to investigate the prognosis and clinical outcome of interventional therapies for CTEPH in the recent era in which different types of interventional therapies, PEA and PTPA, have been available.

## Materials and Methods

### Study Design

This study was conducted based on retrospective data with collaboration of three institutions (Kyorin University Hospital, Keio University Hospital, and Fujita Health University Hospital) in Japan. One hundred and thirty-six patients with CTEPH who attended Kyorin University Hospital or Keio University Hospital, Japan from January 2000 to April 2013 were included. Importantly, PEA had been the only available interventional therapy until 2008 s, and PTPA was started in our institutions in January 2009. Both PTPA and PEA have been proposed as potential candidates since 2009 with consideration of their benefits/risks and possible complications. Thus, a new era of therapeutic strategies for CTEPH by both of PEA and PTPA has been started at 2009 in our institutions.

These patients were diagnosed with CTEPH by demonstration of organized pulmonary thromboembolism using contrast-enhanced lung computed tomography, perfusion lung scintigraphy, and pulmonary angiography, and pulmonary arterial hypertension, pulmonary disease, left heart abnormality, and other systemic diseases had been excluded by blood tests, pulmonary function tests, and echocardiography.

Twenty-nine patients (14 patents between 2000 and 2008, and 15 between 2009 and 2013) received only medical treatment without interventional therapy (Drug-group) because of refusal to have interventional therapy or peripheral location of lesions. Medications consisted of anticoagulants and pulmonary vasodilators such as prostanoids including beraprost, phosphodiesterase-5 inhibitors including sildenafil and tadalafil, and endothelin-receptor antagonists, bosentan and ambrisentan. Thirty-nine patients (38 patients between 2000 and 2008, and 1 between 2009 and 2013) underwent PEA at a Japanese institution of expertise, Fujita Health University Hospital (PEA-group). Sixty-eight patients underwent PTPA, starting in January 2009, at Keio University Hospital or Kyorin University Hospital (PTPA-group). All patients provided written informed consent, and the performance of PTPA and analysis of clinical data performed in the present study were approved by the institutional review boards of Kyorin University Hospital and Keio University Hospital.

### Examinations

All patients underwent right-sided heart catheterization at diagnosis and thereafter almost every year. Right atrial pressure (RAP), pulmonary arterial pressure (PAP), and pulmonary arterial wedge pressure (PAWP) were measured by right-sided heart catheterization. Cardiac output was determined by the Fick technique using assumed oxygen consumption. Cardiac index was calculated by dividing cardiac output by body surface area. Total pulmonary resistance (TPR) was calculated by dividing of mean PAP by cardiac output. Six-minute-walk distance (6MWD) and plasma B-type natriuretic peptide (BNP) level were measured one day before right-sided heart catheterization.

### Selection Criteria for Potential Therapeutic Strategies of Medical Therapy, PTPA, and PEA

The patients were selected as potential candidates for interventional therapies such as PTPA and PEA based on the following criteria; 1) mean PAP more than 30 mmHg or TPR more than 3.75 Wood units, 2) New York Heart Association (NYHA) functional class II or more, and 3) patients who understood the interventional procedures and possible complications, and gave informed consent of their own free will. Meanwhile, the exclusion criteria for interventional therapies were active infectious disease and/or serious co-morbidity such as chronic obstructive pulmonary disease (stage IV), hepatic disease, kidney disease (less than 30% of creatinine clearance), hemorrhagic tendency (less than 5.0×10^4^/µL of platelet level), or poorly-controlled diabetes mellitus or hypertension. PTPA was excluded in patients unable to lie on the treatment table during the procedure.

The patients in the Drug-group included those who did not fulfill the above-mentioned inclusion criteria for the interventional therapies and those who did not want to undergo interventional therapy.

PTPA targets partially the same lesions (segmental and subsegmental lesions) as PEA. Thus, the patients treated with PTPA were those who rejected PEA or for whom we suggested PTPA was more appropriate than PEA because of their advanced age, poor physical condition, or high risk of PEA with right heart failure and a lot of comorbidities. PEA was strongly recommended when the main lesions were located in the central parts of the pulmonary arteries. In the patients who had relatively severe hemodynamic severity but had discrepant angiographic less severe lesions, we considered the possibility that the vasculitis or inflammation of small vessels or microvascular vessels were comorbid disease or, in some cases, main disease. If PEA is performed for those patients, persistent pulmonary hypertension will be expected. Therefore, PTPA was selected for those patients, and balloon dilatation for some target lesions that could be angiographically detected was performed with the aim of improvement of hemodynamics as much as possible.

The procedures for PTPA including selection of the target vessels are described in our previous reports [20], [21]. In PTPA procedure, the lesion types other than pouch defect and complete occlusion, such as webs and bands, intimal irregularities, and abrupt narrowing, were preferentially selected. There is little information about angiographic peripheral vessel structures in lesions of pouch defect and complete occlusion, and the safeness of wiring should be less than other lesion types. Furthermore, the accessibility in PTPA procedure is high in the lesion types other than pouch defect and complete occlusion. PTPA is performed in staged sessions at intervals of 1 to 2 weeks, because of the risk of acute pulmonary edema if excessive target lesions are vascularized at one session and because of limitations in X-ray exposure and the amount of contrast dye used at each session. The targeted values of X-Ray exposure, fluro times, and amount of contrast material in each PTPA session were within 1000 mGy, 60 min, and 300 ml, respectively. Furthermore, we recently demonstrated the usefulness of pulmonary edema predictive scoring index (PEPSI) as a promising predictor to avoid significant reperfusion pulmonary edema [21]. If the endpoint per each PTPA session is determined by setting in less than 35.4 scores of PEPSI, the negative predictive value of reperfusion pulmonary edema is more than 90%. Therefore, the stop of PTPA sessions was also decided in accordance with scores of PEPSI in our recent PTPA sessions. The average number of total sessions per patient in the PTPA-group was 2.5±1.4 (total session number: 178).

### Definition of Endpoints to Evaluate Outcomes of Two Interventional Therapies

To compare the clinical outcomes of the two interventional therapies, the endpoints were defined as 1) cardiac death, 2) right heart failure, and 3) re-vascularization. Right heart failure was defined as the need for hospitalization of the patient for treatment. The necessity for re-vascularization after each interventional therapy was judged when the patient had residual pulmonary vascular resistance of over 6.25 wood units (about 500 dynes.sec.cm^−5^) at more than 3 months after each interventional therapy (for PTPA, more than 3 months after final PTPA session) in accordance with a recently published report [19], and desired additional interventional therapy. They were treated with PTPA as additional re-vascularization therapy. PEA was excluded as a choice for re-vascularization because repeated PEA has a high operative risk.

### Statistical Analysis

Significant differences between the two groups were determined using Mann-Whitney U test or Wilcoxon's matched-pairs signed rank test, as appropriate. Differences in frequencies were analyzed using Fisher's exact probability test.

The outcome regarding cardiac death from the time of diagnosis to April 2013 was compared between patients in the Drug-group and interventional therapy groups (PEA-group plus PTPA-group) by Kaplan-Meier method.

To compare the outcomes of the two interventional therapies, the occurrence of the endpoints from the date of interventional therapy to April 2013 (or, to additional PTPA in cases of re-vascularization) was compared between patients in the PEA-group and PTPA-group by Kaplan-Meier method. The degree of improvement in hemodynamics, 6MWD, and BNP level from the time of interventional therapy to the latest follow-up (or, to the latest follow-up before additional PTPA in cases of re-vascularization) was also compared between the two groups by two-way repeated-measures analysis of variance and Sidak's multiple comparisons test. Univariate analysis based on the log-rank test was used to examine the relationship between occurrence of the endpoints and each interventional therapy, and the results were expressed as hazard ratios with 95% confidence intervals (CI).

All data are presented as mean ± standard deviation. A value of p<0.05 was considered statistically significant.

## Results

### Outcome of Drug-group and Interventions-group

**Figure 1:**
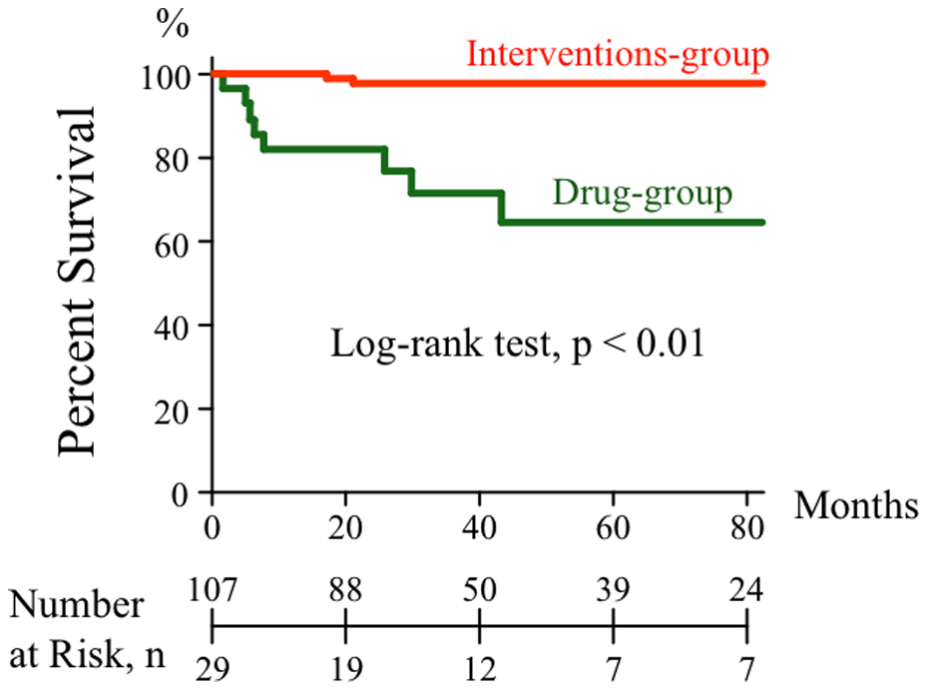
Kaplan-Meier curves showing outcome from diagnosis. Curves show outcome in patients treated with medical therapy alone (Drug-group) (green line) and patients treated with interventional therapy (Interventions-group) (orange line). Patients in the Interventions-group were treated with pulmonary endarterectomy (PEA) and/or percutaneous transluminal pulmonary angioplasty (PTPA). This analysis shows a significantly better outcome in the Interventions-group than in the Drug-group (log-rank test, p<0.01).

Table 1 shows the baseline characteristics at the time of diagnosis. Age, gender, clinical severity according to NYHA functional class, exercise capacity according to 6MWD, and the ratio of administration of specific pulmonary vasodilators were not significantly different between patients in the Drug-group and patients treated with interventional therapy, PEA and/or PTPA (Interventions-group). However, hemodynamic parameters, such as mean RAP, mean PAP, and TPR, and BNP level in the Interventions-group were significantly more severe than those in the Drug-group. In the observation period of 49.3±38.4 months in 136 total patients (41.6±41.1 vs. 51.3±37.6 months in Drug-group vs. Interventions-group, p = 0.0739), the outcome of patients in the Interventions-group was much better than that in the Drug-group (5-year survival 98% vs. 64%, p<0.0001; hazard ratio [95%CI] 0.057 [0.003–0.085] vs. 17.5 [11.8–286.6]), suggesting that the interventional therapies were more effective than medical therapy (Figure 1). Furthermore, between 2000 and 2008, the outcome of patients in the PEA-group was much better than that in the Drug-group (5-year survival rate, 97% vs. 51%, p = 0.0002; hazard ratio [95%CI] 0.057 [0.008–0.231] vs. 17.5 [4.3–129.4]) in the observation period of 42.6±31.0 months in 52 patients [38.1±34.0 vs. 44.3±30.1 months in the Drug-group (n = 14) vs. the PEA-group (n = 38), p = 0.3389].

**Table 1:** Baseline Characteristics at Diagnosis.

Variables	All patients (n = 136)	Drug-group (n = 29)	Interventions-group (n = 107)	p value for Drug-group vs. Interventions-group
Age, y	58±13	60±16	57±13	p = 0.1898
Sex, female/male, n	37/99	9/20	28/79	p = 0.6409
NYHA functional class, I/II/III/IV, n	0/33/87/17	0/17/10/2	0/16/76/15	p<0.0001
Mean RAP, mmHg	6.5±4.0	5.4±3.6	6.8±4.1	p = 0.0391
Mean PAP, mmHg	45.5±10.8	38.2±10.3	47.5±10.2	p<0.0001
TPR, wood units	15.1±7.3	12.4±7.7	15.8±7.1	p = 0.0040
Cardiac index, L/min/m^2^	2.2±0.7	2.4±0.6	2.2±0.7	p = 0.0709
BNP, pg/mL	301±337 (n = 128)	251±444 (n = 29)	316±299 (n = 99)	p = 0.0009
6MWD, m	345±128 (n = 88)	406±103 (n = 15)	332±130 (n = 73)	p = 0.0546
Administration of specific pulmonary vasodilators	115 (85%)	21 (72%)	94 (88%)	p = 0.0774
Prostanoids	75 (55%)	11 (38%)	64 (60%)	p = 0.0570
PDE-5 inhibitors	89 (65%)	17 (59%)	71 (66%)	p = 0.5125
ERAs	69 (51%)	10 (34%)	59 (55%)	p = 0.0602

Data show baseline characteristics at time of diagnosis. Drug-group, patients treated with medical therapy alone; Interventions-group, patients treated with pulmonary endarterectomy (PEA) and/or percutaneous transluminal pulmonary angioplasty (PTPA); NYHA, New York Heart Association; RAP, right atrial pressure; PAP, pulmonary arterial pressure; TPR, total pulmonary resistance; BNP, B-type natriuretic peptide; 6MWD, six-minute-walk distance; PDE-5 inhibitor, phosphodiesterase-5 inhibitor; ERA, endothelin-receptor antagonist; NS, not significant (p>0.05).

In the drug-group, follow-up right-heart catheterization at 3 months or more after starting medical therapy was performed in 17 out of 29 patients. In the average observation period of 31.9±32.5 months, all hemodynamic parameters such as mean RAP, mean PAP, TPR, and cardiac index did not significantly improve (mean RAP, 4.6±3.5 mmHg at baseline to 5.6±4.1 mmHg at follow-up, p = 0.2026; mean PAP, 38.4±9.7 mmHg at baseline to 33.8±11.9 mmHg at follow-up, p = 0.1403; TPR, 12.7±8.1 wood units at baseline to 9.3±7.7 wood units at follow-up, p = 0.0984; cardiac index, 2.4±0.7 L/min/m^2^ at baseline to 3.1±1.2 L/min/m^2^ at follow-up, p = 0.0638).

### Comparison of Outcome According to a Shift in Clinical Practice Occurred in 2009

Importantly, all but one patient underwent PTPA in the interventional group since 2009, suggesting a shift in clinical practice occurred in 2009 in proposing PTPA instead of PEA for inoperable but also operable patients. Therefore, the comparison of the results between two different periods of CTEPH management (2000–2008 vs. 2009–2013) characterized by a shift in clinical practice (PTPA availability) was analyzed. There was no significantly different between the outcome of patients diagnosed in 2000–2008 [n = 63; Drug-group, n = 14 (22.2%) + Interventions-group, n = 49 (77.8%)] vs. that in 2009–2013 [n = 73; Drug-group, n = 15 (20.5%) + Interventions-group, n = 58 (79.5%)] (5-year survival rate, 88.6% vs. 94.9%, p = 0.3918; hazard ratio [95%CI] 1.748 [0.506–6.198] vs. 0.572 [0.161–1.976]).

### Morbidity, Mortality, and Complications of Each Interventional Procedure

Total 213 PTPA sessions was performed in 68 patients of PTPA-group. One patient had the comorbidities of liver dysfunction and renal dysfunction with severe right heart failure before PTPA, as described in another our report [27]. No sessions were failed-out. Clinically-significant reperfusion pulmonary edema occurred in 15 (7.0%) sessions, 5 (2.3%) sessions accompanied with hemosputum which spontaneously stopped later, 7 (3.3%) sessions with hemoptysis which needed long-time ballooning to stop, 5 (2.3%) sessions with dissection of target vessels without perforation, and 2 (0.9%) sessions with wiring perforations, in which one case was recovered after coiling and another case leaded to death. Thus, the mortality rate was 1.47% (1 out of 68 patients).

In the PEA-group, no patients had the comorbidities such as COPD, CRF or hemorrhagic tendency before PEA. With regards to complications of PEA, reperfusion pulmonary edema, pulmonary hemorrhage, low output syndrome, and pneumothorax were 5 (12.8%), 3 (7.7%), 3 (7.7%), and 1 (2.6%), respectively. One patient in the PEA-group became shock state just before PEA, so the patient underwent emergent PEA, but died after 4 days. Therefore, the perioperative mortality rate of PEA was 2.6% in this study.

### Hemodynamics in PTPA-group and PEA-group

We consecutively investigated the clinical outcome with interventional therapies. Table 2 shows the baseline characteristics just before each interventional therapy. Distribution of gender, clinical severity according to NYHA functional class, RAP, TPR, and exercise capacity according to 6MWD were not significantly different between the PTPA-group and the PEA-group. However, age was significantly older in the PTPA-group than in the PEA-group, and hemodynamic parameters in the PEA-group except mean RAP and TPR were significantly more severe in the PTPA-group. These findings could suggest that PEA tended to be performed in younger patients with more severe baseline hemodynamics because PEA is more invasive than PTPA.

**Table 2:** Characteristics before Interventional Therapies.

Variables	PTPA-group (n = 68)	PEA-group (n = 39)	p value for PTPA-group vs. PEA-group
Age, y	62±14	53±10	p<0.0001
Sex, female/male, n	16/52	12/27	p = 0.4944
NYHA functional class			p = 0.0522
Class I, n	0	0	
Class II, n	14	2	
Class III, n	47	29	
Class IV, n	7	8	
Mean RAP, mmHg	5.4±3.6	6.6±3.2 (n = 37)	p = 0.0510
Mean PAP, mmHg	42.1±9.9	52.4±9.7	p<0.0001
TPR, wood units	14.8±6.6	17.6±7.6	p = 0.0574
Cardiac index, L/min/m^2^	2.5±0.6	2.1±0.6	p = 0.0008
BNP, pg/mL	202±233	395±274 (n = 31)	p<0.0001
6MWD, m	342±129 (n = 61)	283±129 (n = 12)	p = 0.2045

Data are values just before each interventional therapy. PTPA-group, patients treated with PTPA; PEA-group, patients treated with PEA. Other abbreviations are defined in Table 1.

**Figure 2:**
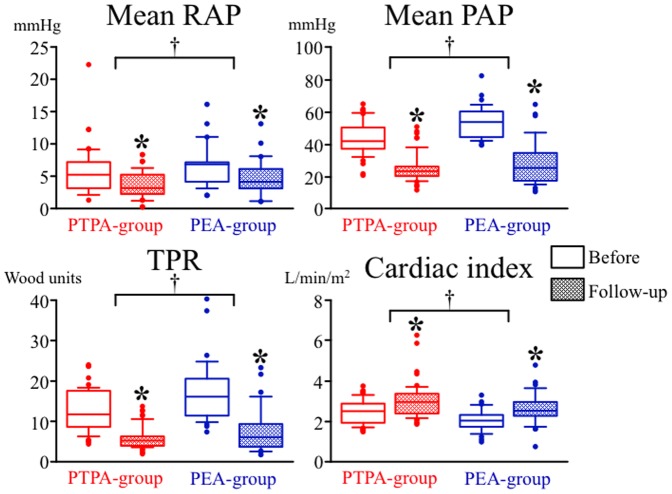
Hemodynamic changes in patients treated with PTPA (PTPA-group) (red boxes) and in patients with PEA (PEA-group) (blue boxes). Open boxes indicate data before each therapy and solid boxes indicate data at follow-up. Data are shown as boxplot distributions. *: statistically significant (p<0.05) difference between before and after each therapy; †: statistically significant (p<0.05) difference between changes of variables in the two groups; RAP: right atrial pressure; PAP: pulmonary arterial pressure; TPR: total pulmonary resistance.

Follow-up right-heart catheterization at 3 months or more after the final interventions was performed in 54 patients in the PTPA-group and 34 in the PEA groups. In the average observation period of 17.4±9.3 in the PTPA-group vs. 74.7±32.3 months in the PEA-group (p<0.0001), all hemodynamic parameters such as mean RAP, mean PAP, TPR, and cardiac index significantly improved in both the PTPA-group and PEA-group (Figure 2). By two-way ANOVA analysis, mean PAP (from 53.1 to 27.9 mmHg) and TPR (from 17.5 to 7.5 wood units) in the PEA-group were improved more than in the PTPA-group (from 42.9 to 25.0 mmHg in mean PAP, from 12.5 to 5.8 wood units in TPR) (mean PAP, p = 0.0004; TPR, p = 0.0001), because these parameters before the procedures were significantly higher in the PEA-group than in the PTPA-group, but there was no significant difference in these post-procedure data between in the two groups. Meanwhile, mean RAP and cardiac index in the PTPA-group were improved more than those in the PEA-group.

### Symptoms, Exercise Capacity, and BNP in PTPA-group and PEA-group

**Figure 3:**
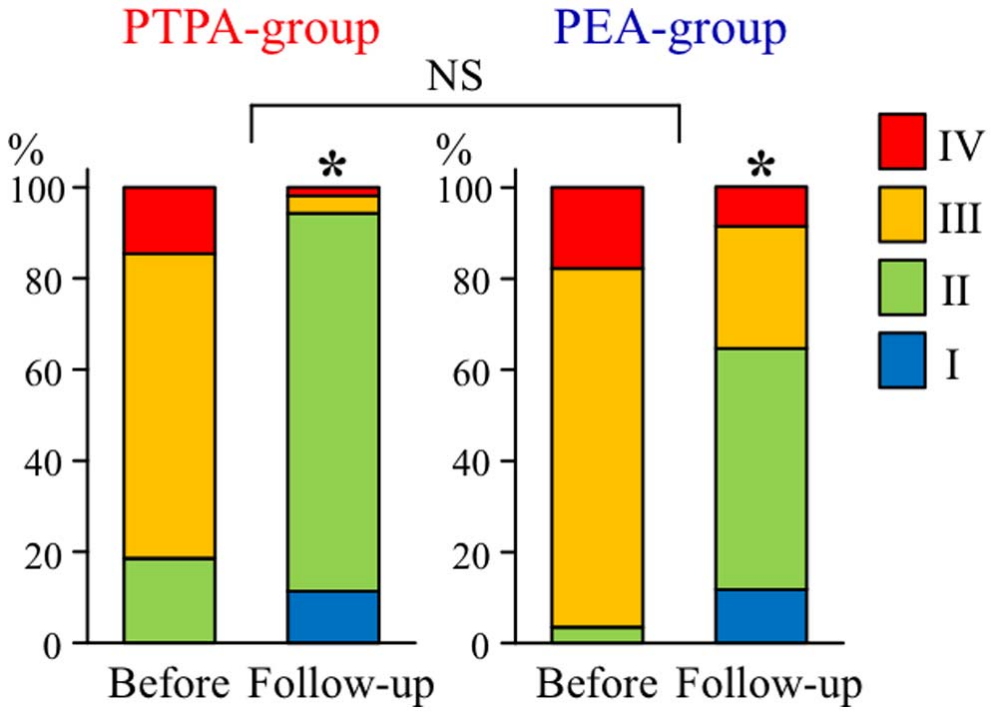
Changes of distribution of New York Heart Association functional class in PTPA-group and PEA-group. *: statistically significant (p<0.05) difference between before and after each therapy; NS, not significant (p>0.05).

**Figure 4:**
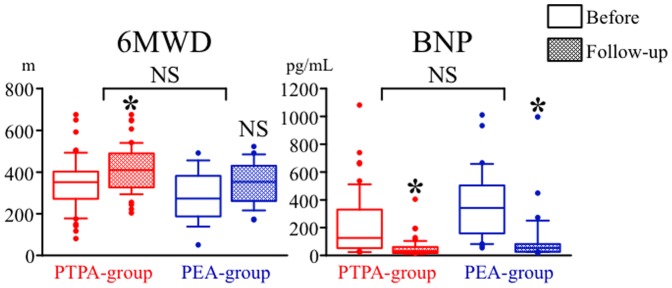
Changes in 6-minute-walk distance (6MWD) and plasma B-type natriuretic peptide (BNP) level in PTPA-group (red boxes) and PEA-group (blue boxes). Open boxes indicate data before each therapy and solid boxes at follow-up. Data are shown as boxplot distributions. *: statistically significant (p<0.05) difference between before and after each therapy.

Symptoms evaluated in terms of NYHA functional class also significantly improved in both the PTPA-group and PEA-group, and there was no significant difference in the degree of improvement in NYHA functional class between the two groups (Figure 3). Exercise capacity evaluated by 6MWD significantly improved in the PTPA-group (349±130 m before PTPA to 424±111 m at follow-up, p<0.0001, n = 45), but did not improve in the PEA-group (326±116 m before PEA to 353±93 m at follow-up, p = 0.3125, n = 11), and there was no significant difference in the degree of improvement in 6MWD between the two groups (Figure 4). BNP, an indicator of right-sided heart overload, also significantly improved in both the PTPA-group (209±223 pg/mL before PTPA to 45±61 pg/mL at follow-up, p<0.0001, n = 54) and PEA-group (374±279 pg/mL before PEA to 108±219 pg/mL at follow-up, p = 0.0004, n = 23), and there was no significant difference in the degree of improvement in BNP between the two groups.

### Endpoints in PTPA-group and PEA-group

**Figure 5:**
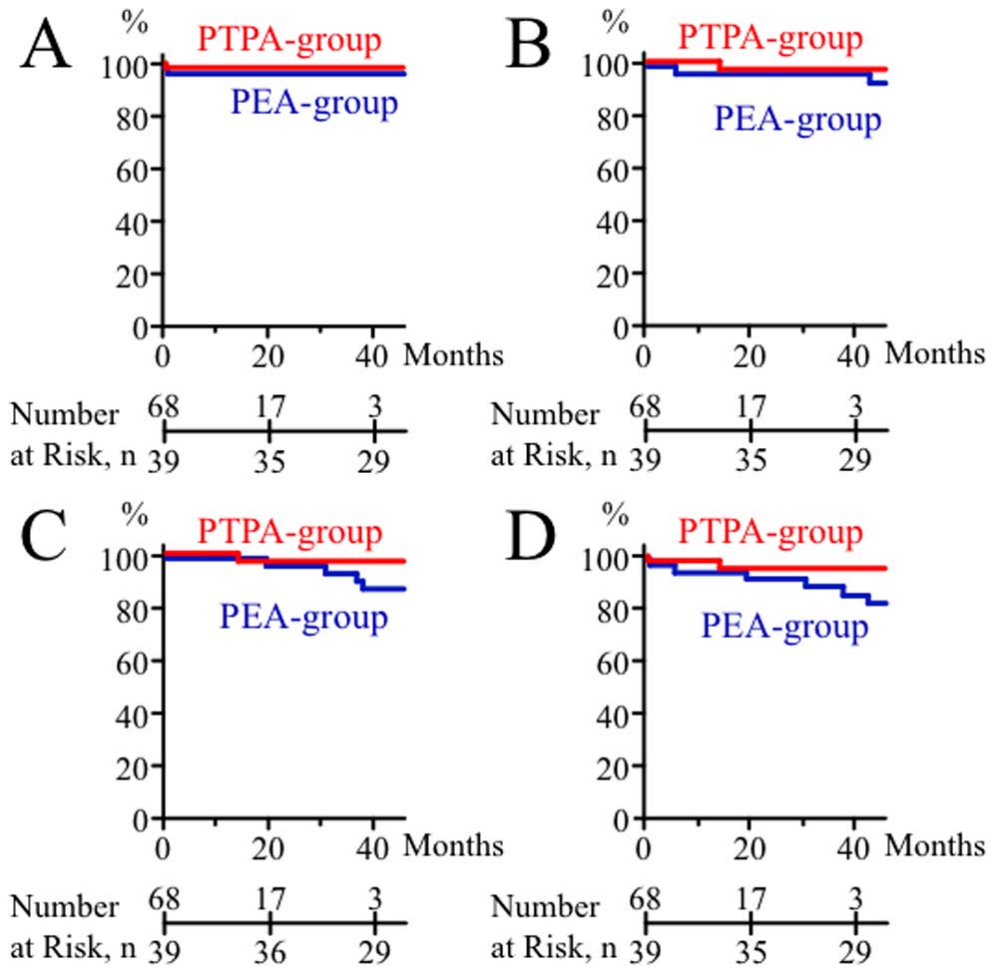
Kaplan-Meier curves to evaluate outcomes of two interventional therapies. Curves show survival rate (**A**), rate of exacerbation of right heart failure (**B**), rate of additional re-vascularization (**C**), and these composite endpoints (**D**) from the time when each interventional therapy (PTPA or PEA) was performed in patients in PTPA-group (red line) and PEA-group (blue line).

During the follow-up period of 33.2±34.3 months in 136 total patients (14.3±10.4 in PTPA-group vs. 66.1±36.7 months in PEA-group, p<0.0001), one patient in the PTPA-group (1.5%) and 2 patients in the PEA-group (5.1%) died. One patient in the PTPA-group had a wiring perforation as a complication of PTPA procedure and died 2 days after the procedure. One patient in the PEA-group died 4 days after PEA, and another patient in the PEA-group had persistent pulmonary hypertension after incomplete endarterectomy and died due to exacerbation of right heart failure before starting time of PTPA at 2009. Survival analysis by Kaplan-Meier method demonstrated no statistically significant difference in survival between the PTPA-group and PEA-group (2-year survival 98.5% vs. 97.4%, p = 0.7275; hazard ratio [95%CI] 0.59 [0.03–10.15] vs. 1.69 [0.10–30.52]) (Figure 5A).

Regarding right heart failure, 1 patient in the PTPA-group (1.5%) and 4 patients in the PEA-group (10.3%) needed in-hospital treatment for exacerbation of right heart failure during the follow-up period. Rate analysis by Kaplan-Meier method demonstrated no significant difference between the PTPA-group and PEA-group (2.9% vs. 2.6% 2-year in-hospital treatment rate after each interventional therapy, p = 0.7920; hazard ratio [95%CI] 0.89 [0.06–14.32] vs. 1.12 [0.07–18.01]) (Figure 5B).

Furthermore, 10 out of 39 patients in the PEA-group (25.6%) needed re-vascularization during the follow-up period and underwent additional PTPA as re-vascularization therapy. Follow-up right-heart catheterization at 3 months or more after the final PTPA was performed in 9 out of these 10 patients who underwent PTPA after PEA with persistent pulmonary hypertension due to incomplete endarterectomy. In the average observation period of 20.3±16.5 months, hemodynamic parameters such as mean PAP (41.9±11.8 mmHg before PTPA to 25.0±6.1 mmHg at follow-up, p = 0.0039) and TPR (11.4±5.3 wood units before PTPA to 6.2±2.6 wood units at follow-up, p = 0.0117) significantly improved, but mean RAP (5.1±2.3 mmHg before PTPA to 3.7±1.8 mmHg at follow-up, p = 0.1406) and cardiac index (2.5±0.6 L/min./m^2^ before PTPA to 2.8±0.9 L/min./m^2^ at follow-up, p = 0.6523) did not improved. In contrast, only 1 out of 68 patients in the PTPA-group (1.5%) needed re-vascularization. In this 1 patient, additional PTPA for re-vascularization improved hemodynamics (mean RAP, from 7 to 3 mmHg; mean PAP, from 37 to 23 mmHg; cardiac index, from 3.35 to 3.65 L/min/m^2^; TPR, from 7.2 to 4.2 wood units). No one underwent PEA after failed PTPA. Kaplan-Meier analysis demonstrated no significant difference in re-vascularization rate between the PTPA-group and PEA-group (2-year re-vascularization rate after each interventional therapy, 2.9% vs. 2.8%, p = 0.6498; hazard ratio [95%CI] 1.48 [0.09–25.49] vs. 0.67 [0.04–11.17]) (Figure 5C).

Composite endpoint analysis of the endpoints of cardiac death, right-heart failure exacerbation, and re-vascularization demonstrated no significant difference between the PTPA-group and PEA-group (2-year composite endpoint rate after each interventional therapy, 4.4% vs. 7.8%, p = 0.5412; hazard ratio [95%CI] 0.59 [0.10–3.34] vs. 1.70 [0.30–10.05]) (Figure 5D).

## Discussion

In recent years, several effective vasodilators have been developed for the treatment of pulmonary hypertension, and it is reported that the prognosis of patients with CTEPH has been markedly improved even with only medical therapy [28]. However, interventional therapy with PEA has been definitely identified as a more powerful treatment for CTEPH, with which organic obstruction and stenosis of the pulmonary arteries can be effectively treated. Recently, reports demonstrating effectiveness of balloon-based catheterization angioplasty have been published by Feinstein et al. in 2001 and from a few institutions including ours in the last one to two years [17], [20]–[26]. Therefore, investigation of outcome in the present era, with two interventional therapies of balloon-based angioplasty, PTPA, and PEA, is much more meaningful. In the present study, the outcome was much better in patients treated with interventional therapies (PEA or PTPA or PEA+PTPA) in the observation period of more than 4 years. This finding strongly demonstrates that the recently available interventional therapies definitely improve the prognosis of patients with CTEPH. The effectiveness of PEA to improve the prognosis of CTEPH has already been established, and a previous report demonstrated 1- and 3-year survival of 88 and 76%, respectively, for patients treated with PEA [29]. In our results, the interventional therapies, PEA and PTPA, resulted in a 98% 5-year survival from diagnosis, suggesting the possibility that the prognosis of patients with CTEPH has improved in the recent era, in which both PEA and PTPA can be chosen, compared to that in the previous era, when PEA was the only choice of interventional therapy.

Moreover, we compared the clinical outcomes of each interventional therapy. The present study found significant improvement in hemodynamic parameters, exercise capacity indicated by 6MWD, and plasma BNP level after PTPA or PEA. The degree of improvement in hemodynamics after PEA in our results was about a 46% decrease in mean PAP and a 49% decrease in TPR, which are consistent with previous reports [30]–[32]. Furthermore, our results demonstrated that the clinical outcome, indicated by hemodynamics, 6MWD, and BNP after PTPA, was comparable to that after PEA. Importantly, both interventional therapies could improve the hemodynamics to the same levels at follow-up, although the follow-up periods were different in length. Additionally, the improvement in survival and prevention of exacerbation of right heart failure after PTPA were also comparable to those after PEA. Another Japanese institution reported in 2012 that the 2-year survival rate from diagnosis was 100% in a smaller population (n = 12) treated with PTPA [25]. Combined with these findings, it is strongly suggested that PTPA, as well as PEA, is also a promising therapeutic strategy for CTEPH.

Intriguingly, the present study demonstrated that the additional re-vascularization rate was not statistically different after each interventional therapy by Kaplan-Meier analysis during our follow-up period. However, the data suggested that the re-vascularization rate after PEA tended to increase after 3 years or more (Figure 5C), although the precise re-vascularization rate after PTPA needs to be evaluated over a longer observation period in the future. In fact, about one-fourth of patients (25.6%) in the PEA-group needed re-vascularization during the follow-up period and underwent additional PTPA as re-vascularization therapy. Conventionally, residual pulmonary hypertension due to incomplete endarterectomy, inaccessible chronic thromboemboli such as peripheral lesions, or small-vessel arteriopathy has been thought to be a crucial concern in PEA [1]. Residual pulmonary hypertension itself could be an important predictor of late postoperative adverse events [30]. In fact, a previous report demonstrated that over 90% of patients treated with PEA had persistent pulmonary hypertension, defined by both mean PAP of 25 mmHg or greater and pulmonary vascular resistance of 240 dynes.sec.cm^−5^ or greater, at 3 months after PEA [29]. In contrast, PTPA can treat peripheral or distal narrow lesions that cannot be reached by PEA [20], [21], which may be the reason why there was a tendency that the re-vascularization rate after PEA was higher than that after PTPA. In this study, additional PTPA as re-vascularization was effective in patients who had persistent pulmonary hypertension after incomplete endarterectomy, suggesting the usefulness of combination therapy by additional PTPA after PEA.

The first benefit of PTPA is a less-invasive intervention without thoracotomy procedure and systemic anesthesia, and with very low mortality (1.47% in this study). The second benefit of PTPA is promising hemodynamic improvements. In fact, 68% of patients in the PTPA-group attained less than 30 mmHg of mean PAP and 46% escaped from pulmonary hypertension (less than 25 mmHg of mean PAP) by average 3.1±1.5 sessions in this study. Risks and possible complication rates of PTPA should be clinically-critical reperfusion pulmonary edema (occurrence rate: 7.0% of sessions in this study) and pulmonary arterial injuries (3.3% of sessions in this study). Reperfusion pulmonary edema has been considered to be a major complication of PTPA [19]–[21], which may be the main reason why PTPA has not been performed aggressively or prevailed. However, we recently reported the efficacy of pulmonary edema predictive scoring index (PEPSI)-guided PTPA to obtain maximum therapeutic efficacy with minimized risk of reperfusion pulmonary edema [21]. Therefore, realizing the significance of PTPA in the present study and referring to recently clarified findings reported from our institutions and others, will promote the more widespread use of PTPA.

There are several limitations of this study. 1) The comparison between PEA and PTPA is slightly unfair, since PEA patients had more severe disease, with much higher TPR. 2) During an observation period of more than 10 years, only 136 patients are included and only 107 patients underwent interventional therapies. The low number of patients cannot exclude that there is a strong selection bias. 3) Only a limited number of PEA procedures were performed (39 patients in 13 years). 4) The patients in the PEA-group and those in the PTPA-group were also treated with the drugs targeted for pulmonary hypertension. Therefore, the drug effects on the favorable outcomes of these groups cannot be excluded. Additionally, 5) the real new era in this study, in which 3 different types of therapeutic strategies, such as medical therapy, PEA, and PTPA, can be available, has been started from 2009, but only 2 therapeutic strategies such as medical therapy and PEA had been available until 2008. The present study is a retrospective case-control study. But, the comparisons between different periods and those between different patient selections in this study cannot imply the real outcomes of these therapies. Thus, a large-scale prospective study using matched patients should be performed to further elucidate the real clinical outcomes of these therapies.

In conclusion, the patients who underwent interventional therapies had better results than those treated only with drugs. The availability of both of these operative and catheter-based interventional therapies leads us to expect the dawn of a new era of therapeutic strategies for CTEPH.
